# Defining Patient-Level Molecular Heterogeneity in Psoriasis Vulgaris Based on Single-Cell Transcriptomics

**DOI:** 10.3389/fimmu.2022.842651

**Published:** 2022-07-26

**Authors:** Yale Liu, Hao Wang, Christopher Cook, Mark A. Taylor, Jeffrey P. North, Ashley Hailer, Yanhong Shou, Arsil Sadik, Esther Kim, Elizabeth Purdom, Jeffrey B. Cheng, Raymond J. Cho

**Affiliations:** ^1^ Department of Dermatology, The Second Affiliated Hospital of Xi’an Jiaotong University, Xi’an, China; ^2^ Department of Dermatology, Veterans Affairs Medical Center, San Francisco, CA, United States; ^3^ Department of Dermatology, University of California, San Francisco, San Francisco, CA, United States; ^4^ Department of Statistics, University of California, Berkeley, Berkeley, CA, United States; ^5^ Clinical Research Centre, Medical University of Białystok, Białystok, Poland; ^6^ Department of Dermatology, Huashan Hospital, Fudan University, Shanghai, China; ^7^ Department of Plastic Surgery, University of California, San Francisco, San Francisco, CA, United States

**Keywords:** single-cell RNA-sequencing, psoriasis vulgaris, heterogeneity, cytoskeleton, chromatin

## Abstract

Identifying genetic variation underlying human diseases establishes targets for therapeutic development and helps tailor treatments to individual patients. Large-scale transcriptomic profiling has extended the study of such molecular heterogeneity between patients to somatic tissues. However, the lower resolution of bulk RNA profiling, especially in a complex, composite tissue such as the skin, has limited its success. Here we demonstrate approaches to interrogate patient-level molecular variance in a chronic skin inflammatory disease, psoriasis vulgaris, leveraging single-cell RNA-sequencing of CD45^+^ cells isolated from active lesions. Highly psoriasis-specific transcriptional abnormalities display greater than average inter-individual variance, nominating them as potential sources of clinical heterogeneity. We find that one of these chemokines, *CXCL13*, demonstrates significant correlation with severity of lesions within our patient series. Our analyses also establish that genes elevated in psoriatic skin-resident memory T cells are enriched for programs orchestrating chromatin and CDC42-dependent cytoskeleton remodeling, specific components of which are distinctly correlated with and against Th17 identity on a single-cell level. Collectively, these analyses describe systematic means to dissect cell type- and patient-level differences in cutaneous psoriasis using high-resolution transcriptional profiles of human inflammatory disease.

## Introduction

Individuals with psoriasis vulgaris broadly share cutaneous features such as erythema, micaceous scale, and induction at skin sites affected by friction. While the role of Th17 cell-produced cytokines such as *IL17F* and *IL26* in generating these phenotypes is well-established (1, 2), the distinctive morphology of these lesions suggests that a broad array of yet uncharacterized downstream effector genes are also specific to and shared by psoriatic lesions. Conversely, individual cases of psoriasis can markedly differ in presentation. Each patient develops lesions in distinct anatomic patterns, for example whether the scalp or intertriginous skin is involved, and lesional itch is also highly variable. These patterns of difference must reflect underlying molecular heterogeneity, potentially related to other clinical features such as involvement of other organ systems (*e.g.* psoriatic arthritis) or response to the many pathway-targeting agents now available for treatment. One well-established example is the involvement of germline *CARD14* variants in psoriasis patients with presentations overlapping with or including the disease state pityriasis rubra pilaris (3). However, many more yet undiscovered gene-based variances on the genetic and epigenetic level are likely to determine an individual patient’s clinical state.

In the past, bulk RNA-sequencing of tissue obtained from lesional skin has been used to detect and define such commonalities and differences, enabling rough estimates of genetic variance in both psoriasis vulgaris and atopic dermatitis (4–6). Such approaches, however, conflate gene expression values from many different immune and stromal cell types, providing relatively crude estimates of genetic similarity and variance. The recent emergence of single-cell profiling technologies, such as single cell RNA sequencing (scRNA-seq) and Cellular Indexing of Transcriptomes and Epitopes (CITE-seq) (7), offers the ability to compare instances of chronic skin inflammatory disease with far greater resolution. We can now ask, for example, what molecular abnormalities are shared by effector immune cells in most psoriasis patients, regardless of clinical presentation? Such recurrent derangements might suggest treatment of psoriasis with existing drugs affecting those targets. Alternatively, certain molecular abnormalities are likely to be found in only a subset of individual cases, nominating them as candidates for specific targeted therapies.

To formally deconstruct discrete levels of molecular heterogeneity underlying cutaneous psoriatic inflammation, we analyzed data from a recent study profiling 8 psoriasis samples and 7 normal controls using single-cell RNAseq (scRNA-seq) and CITE-seq based on the 10X Genomics Chromium platform (8). We intended to develop and test approaches to scRNA-seq datasets profiling chronic inflammatory disease that could be practically and widely applied as similar datasets become published.

## Materials and Methods

### Clinical Sample Acquisition

Patient recruitment and methods are detailed in our companion publication (8). Briefly, written informed consent was procured from donors providing both normal and psoriatic lesional skin under protocols approved by the University of California, San Francisco Institutional Review Board. Full thickness punch biopsies (6 mm) were obtained from psoriasis lesions; discards from abdominoplasties and mammoplasties were used as normal controls. All patients had not used topical immunosuppressives for at least 2 weeks before biopsy. All patients were naïve to targeted biologic medications or disease-modifying non-steroidal agents except for Patient 5, who was under systemic immunosuppression following a liver transplant. Clinical details of psoriasis samples in our series are described in **Supplementary Table 1**.

### CD45^+^ Immune Cell Isolation, Single-Cell RNA-seq and CITE-Seq Profiling, and Data Processing

Details of skin biopsy sample processing, CD45^+^ immune cell isolation, 10X Genomics 3’ scRNA-seq and CITE-seq library preparation, and data analysis are further described in a recent prior publication (8). Briefly, we initially performed high-resolution clustering and eliminated populations corresponding to non-immune and low quality cells (mitochondrial genes percentage <20%, 100 < nFeatures < 6000). With the remaining cells, we performed unsupervised clustering with the following in Seurat (15 harmonies to run UMAP() and 1.0 resolution for FindClusters()to obtain the final 20 clusters used in this analysis. Marker transcripts for each cluster were identified using the *FindAllMarkers* function in Seurat (results are in **Supplementary Table 2**). Cluster identities were then manually annotated based on canonical immune cell population markers.

### Sample-Specific Differential Gene Expression Identification, Dispersion Score Calculation, and Metascape Analysis

We created pseudo-bulk counts for each patient for the cells that were mapped to CD45^+^ cell subpopulations using the package *muscat* (9) in Bioconductor. The *muscat* method aggregates the single-cell data at the cluster-sample level to create pseudo-bulk data and then applies the methods of *edgeR* (10) to pseudo-bulk calculations to identify DEGs between normal and psoriasis samples (volcano plot, **Figure 2A**). To calculate dispersion values of the psoriasis samples, we applied the function estimateTagwiseDisp from the *edgeR* package in Bioconductor to the pseudo-bulk counts from the psoriasis samples. To identify abnormally elevated, functionally related gene sets (*e.g.* Gene Ontology (GO), Reactome) in Trm2, we applied the Metascape package (11, 12) to significant DEGs identified by FindMarkers() in comparison to grouped healthy controls (*p* < 0.05).

### Normalization of T Cell Number Expressing Specific Immune Cell DEGs for Each Psoriasis Biopsy

Although all psoriasis biopsies were 0.6 cm in diameter, different proportions of isolated cells were scRNA-seq processed for each sample. To determine the number of T cells expressing each DEG in each biopsy, we took the assessed number of expressing T cells for a given DEG and adjusted by total number of CD45^+^ cells obtained from each biopsy/total cell number processed in Seurat.

### Statistical Correlation Analysis

Gene values were batch-corrected at the sample level using the CPCA method in the R package iCellR; missing gene values were independently imputed within inflamed and unflamed states of sample-aligned matrices using the PCA method in iCellR/run.impute. Resulting matrices were then used for the correlation matrix. Rstudio v.1.4.1717 and GraphPad Prism (version 8.0; GraphPad Software, La Jolla, California) were used for statistical analysis and heatmap generation. Pearson correlation coefficients were calculated for gene-gene comparisons using the R function cor(). Adjusted *p* < 0.05 was considered significant for Seurat-based analyses, while *p* < 0.05 was used for other analyses.

## Results

### scRNA-Seq-Based Classification of Major T and Antigen-Presenting Cell Types Isolated From Psoriatic and Normal, Uninflamed Skin

We focused on 7 normal and 8 psoriasis samples from the Liu et al. study (8) (**Supplementary Table 1**). Diagnoses were based on clinical evaluation by a board-certified dermatologist and confirmed by formal histopathological reading. Six of eight patients were judged to have moderate to severe disease based on Psoriasis Area and Severity Index (PASI) scores and two (Patients 2 and 5) were in the mild range (**Supplementary Table 1**). The only patient known to be taking systemic immunosuppressive treatments within 4 weeks of biopsy was Patient 5, who was maintained daily on 4 mg of tacrolimus and 1250 mg of mycophenolate mofetil following a liver transplant. Normal controls were taken from discarded tissue obtained from mammoplasties and abdominoplasties.

Briefly, skin biopsies were enzymatically digested and flow sorted for live CD45^+^ cells, which were then subjected to Chromium 3’ single cell RNA-seq and CITE-seq protein epitope sequencing. Single-cell transcriptomic data was obtained from an average of ~5,200 single cells per sample after eliminating doublets, poor-quality, as well as non-immune cells. To classify cells, a graph-based clustering approach using Louvain community detection-based modularity optimization, available in the Seurat package, was utilized.

**Figure 1:**
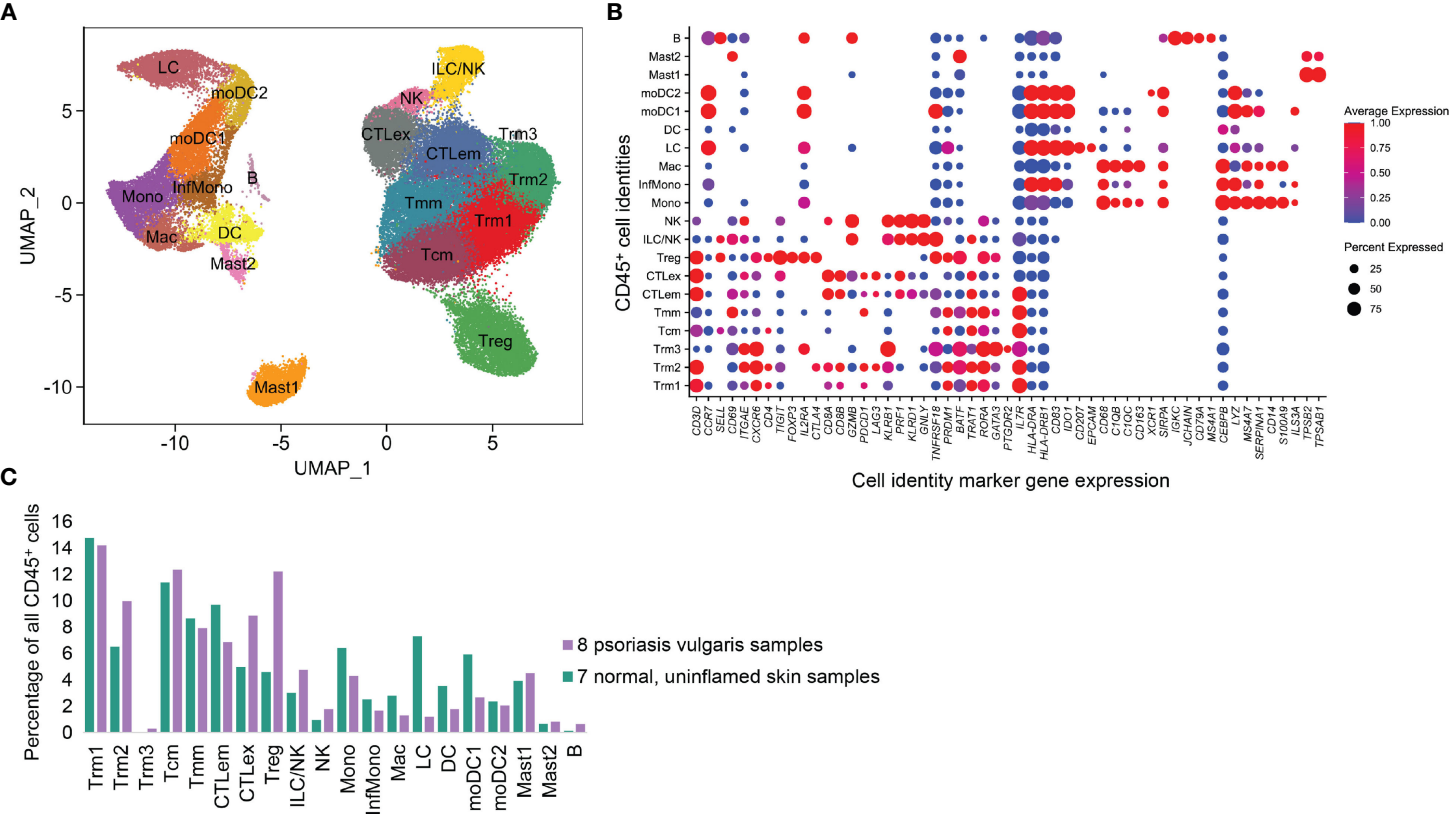
CD45^+^ immune cell types identified from 8 psoriasis vulgaris lesions and 7 normal skin samples. **(A)** UMAP representation of 11 T cell and 9 APC classes based on scRNA-seq transcriptional data, in which each point represents a single cell. **(B)** Expression of critical marker transcripts distinguishing immune cell classes. **(C)** Proportion of each immune class in total CD45^+^ cell populations.

We obtained 20 cell types based on previously described unsupervised clustering approaches (8). Robust representation of each sample was observed (**Supplementary Data 1**). As shown in **Figure 1**, the most upregulated transcripts in each cluster (so-called marker genes) define a central memory cell population (*CD3D^+^/CCR7* ^+^/*SELL* ^+^/*KLF2* ^+^) we call Tcm, as well as a migratory memory class Tmm (*CD3D^+^/CCR7* ^+^/*SELL* ^-^). Based on expression of *ITGAE* (*CD103*), *CXCR6*, and *CD69*, we identified three resident memory populations (Trm1, Trm2, and Trm3). A *CD4* ^+^ regulatory T cell (Treg) population was noted based on the expression of *FOXP3, TIGIT, CTLA4, IL2RA (CD25)*, and *IKZF2* (Helios).

Two cytotoxic (*CD8A^+^CD8B^+^*) T cell clusters expressing *CCL5, GZMB*, and *NKG7* were identified. One we annotated as cytotoxic effector memory cells *(*CTLem*)* due to expression of effector molecules including *TNFRSF18* and *CD96*, as well as resident markers *CD69* and *ITGAE.* Interestingly, the second cytotoxic T cell population was quantitatively enriched in the psoriasis vs. normal samples and contained elevated canonical exhaustion markers such as *PDCD1* and *LAG3*. Accordingly, this population was classified as exhausted T cells (CTLex). There were also two populations with high *KLRD1^+^*, *GNLY^+^*, *PRF1^+^*, and *GZMB^+^* expression, one with high levels of the CD56 epitope by CITE-seq (NK cells) and the other defined as ILC/NK cells.

Antigen-presenting cell types (APCs) were also classified based on canonical markers. A macrophage population was enriched for *CD68*, *CEBPB*, and *FCER1G*, as well as complement transcripts *C1QB* and *C1QC* and the scavenger receptor *CD163* (Mac). We also examined four monocyte or monocyte-derived cell populations with elevated *MS4A7*, *LYZ*, and *SERPINA1*. There was an inflammatory monocyte (InfMono) population characterized by increased *IL1B* and *IL23A* and another cluster of classical monocytes (Mono) which expressed higher *S100A9* and *CD14*. Two of these clusters also expressed very high MHCII molecule levels (*HLA-DRA*, *HLA-DRB1*) and were identified as monocyte-derived DC (moDC1 and moDC2). A dendritic cell (DC) class (*HLA-DRA* ^+^) was enriched in *CLEC10A*. A population with *EPCAM*, and *CD207* was defined as Langerhans cells (LC). A small population comported with the B cell lineage, with high expression of *IGHG, IGHA, IGKC, JCHAIN, CD19*, and *MA4A2).* Two clusters of Mast cells (Mast) were distinguished by expression of *TPSB2* and *TPSAB1* (Mast1 and Mast2).

### Psoriasis-Specific Transcriptional Abnormalities in Skin-Resident Memory T Cells Show High Patient-Level Variance

We next applied a pseudo-bulk method to identify differentially related genes (DEGs) that distinguished immune cell populations in our 8 psoriasis samples from 7 grouped healthy control biopsies. This approach aggregates scRNA-seq-derived gene counts for each cell subpopulation in each individual sample. Standard bulk mRNA-Seq computational approaches for differential expression were then applied, thereby allowing for patient-level variance to influence the significance of individual DEGs (9). One notable feature of our recent comparisons of psoriasis and other rash types such as atopic dermatitis is that the large majority of psoriasis-specific transcriptional changes are detected in Trm (8). For example, in the Tcm compartment, excluding mitochondrial and ribosomal transcripts, only *KLRB1*, *IL17R*, and *JUN* were expressed at greater than 0.5 logFC in psoriasis compared to normal samples. In Tregs, only *CPM*, *TNFRSF*, *CD7*, *FTH1*, *IL7R*, *MAGEH1*, *MAL*, *TBC1D4*, met these criteria. For APC classes, the far smaller number of cells captured in our CD45^+^ cell-centric approach led to detection of even fewer highly specific DEGs.

Consistent with these recent findings, our pseudo-bulk analysis primarily detected upregulation of Th17 cytokines such as *IL17F* and *IL26*, as well as established psoriasis inflammatory markers such as *IFNG* and *CXCL13* (13), in a skin-resident memory T cell compartment (Trm2). Therefore, we mainly focused on this T cell class for further analysis. Overall, in Trm2 we identified 1,425 transcripts that distinguished psoriasis from healthy controls at a *p* value of < 0.05 (**Supplementary Table 3**).

**Figure 2:**
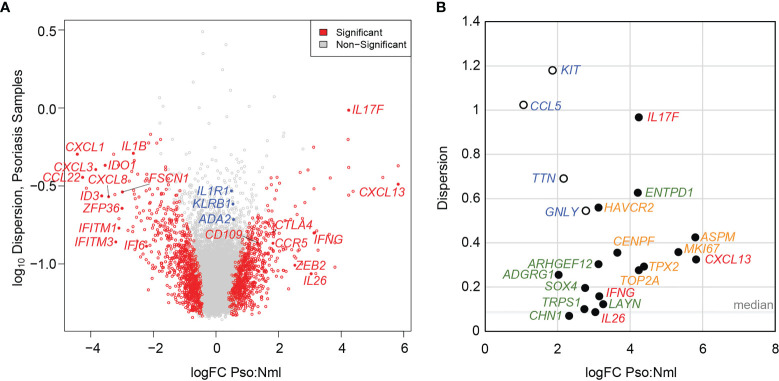
Elevated patient level variance in psoriasis-specific skin-resident memory T cell (Trm2) DEGs. **(A)** Volcano plot showing psoriasis DEGs identified using a pseudo-bulk approach charted as a function of logFC difference from normal, uninflamed cells (*x*-axis) and the log of the dispersion score (a proxy for patient-level variation, *y*-axis). Significant DEGs are shown in red, non-significant DEGs in grey. Labelled in blue are immune activation genes with relatively high dispersion scores, which may have prevented them from reaching statistical significance. **(B)** LogFC (*x*-axis) and dispersion score (*y*-axis) shown for established pathogenic psoriatic cytokines (red), mitotic cell division transcripts (green), psoriasis-specific abnormalities not elevated in atopic dermatitis (orange), and as in **(A)**, immunologically activating DEGs with high end dispersion scores (blue).

Understanding patient-level variance, alongside fold-change magnitudes, is foundational to the conceptualization and use of disease biomarkers. Transcripts that distinguish psoriatic from normal tissue at higher log-fold change and relatively low patient-specific variance may perform well in broad screening efforts. Conversely, DEGs with high patient-level variance should be investigated as possible sources of phenotypic variance between affected individuals. To assess the contribution of patient-level variation to DEG identification in the skin-resident memory T cell population Trm2, we calculated dispersion values using *edgeR* (14) across our dataset. Lower dispersion scores correlate with lesser patient-level variation, which increases the significance of a pseudo-bulk-identified DEGs at a given logFC. **Figure 2A** plots logFC (*x*-axis) and dispersion score (*y*-axis), with transcripts with *p* < 0.05 adjusted value shown in red. In addition to the Th17 cytokines noted above, this representation shows significant elevation of immune activation markers such as *CTLA4*, *CCR5*, *CD109*, and *ZEB2*. We also saw clear suppression of other inflammatory pathways, including the interferon signaling genes *IFITM1*, *IFITM3*, and *IFI6* and the chemokines *CXCL3* and *CXCL8*. This representation also illustrates how greater patient-level variation for a given DEG (higher dispersion score along the *y*-axis) decreases its significance. For example, *IL1R1*, implicated in licensing Th17 cytokine production (15), *ADA2*, an adenosine deaminase central to T cell maturation (16), and the psoriasis-associated CD161 receptor gene *KLRB1 (* 17) show psoriasis-specific elevation in the logFC 0.5 range, but Log_10_ dispersion scores of greater than -1, likely contributing to their failure to reach statistical significance in comparison to healthy control skin-residency T cells (annotated in blue in **Figure 2A**, data in **Supplementary Table 3**).

We more closely examined inter-individual variance in psoriasis DEGs that were identified in the prior analysis as elevated not only relative to normal controls, but also to atopic dermatitis samples, indicating greater disease-specificity (8). Notably, many of these genes showed dispersion scores greater than the median of 0.086 (**Figure 2B**). In fact, for the six psoriasis DEGs with a logFC > 3 and significantly elevated compared to atopic dermatitis, the average dispersion score was 0.484 with a standard deviation of 0.273. In addition to the cytokines noted above such as *IL17F* (0.967), and *CXCL13* (0.325), this set contained identified psoriasis-specific genes with less established functional roles, such as *ARHGEF12* (0.303), *ENTPD1* (0.628), *LAYN* (0.122) and *HAVCR2* (0.560).

Cell cycle transcripts, which are elevated in both psoriasis and atopic dermatitis Trm2 compared to healthy controls, also show higher than median dispersion scores, including *MKI67* (0.358), *TOP2A* (0.276), and *CENPF* (0.356). Similar to **Figure 2A**, **Figure 2B** displays examples of psoriasis-implicated genes whose expression is elevated in Trm2, but whose high inter-individual variance reduces their overall significance level (*i.e. KIT*, *CCL5*, *TTN*, and *GNLY*, blue, open circles).

### *CXCL13* and *CD84* Expression in Cutaneous T Cells Corresponds With Lesional Psoriasis Severity

We were next curious to understand if expression of psoriasis-specific immune DEGs correlated with clinical features such as PASI score. Such relationships might further narrow the search for genetic factors influencing clinical heterogeneity in psoriasis. We chose 16 established immune activation genes from our Trm2 DEGs including *IL17F*, *CXCL13*, *IL26*, *CCR5*, and *CD82* (**Supplementary Table 4**) and quantified the T cells in each sample that detectably expressed each. We normalized these cell numbers between patients by bioinformatically deducing the total number of such cells existing in each sample, based on the total number of CD45^+^ cells obtained from each biopsy, as well as the total number of scRNA-seq profiled cells processed in Seurat (Materials and Methods, **Supplementary Table 4**). To generate accompanying measures of clinical severity, we reasoned that the phenotype of a biopsied and molecularly profiled lesion would be best represented by summing its individual Erythema, Induration, and Desquamation PASI descriptors, rather than the overall patient score, and derived such a lesion-specific severity score for each sample (**Supplementary Table 1**).

**Figure 3:**
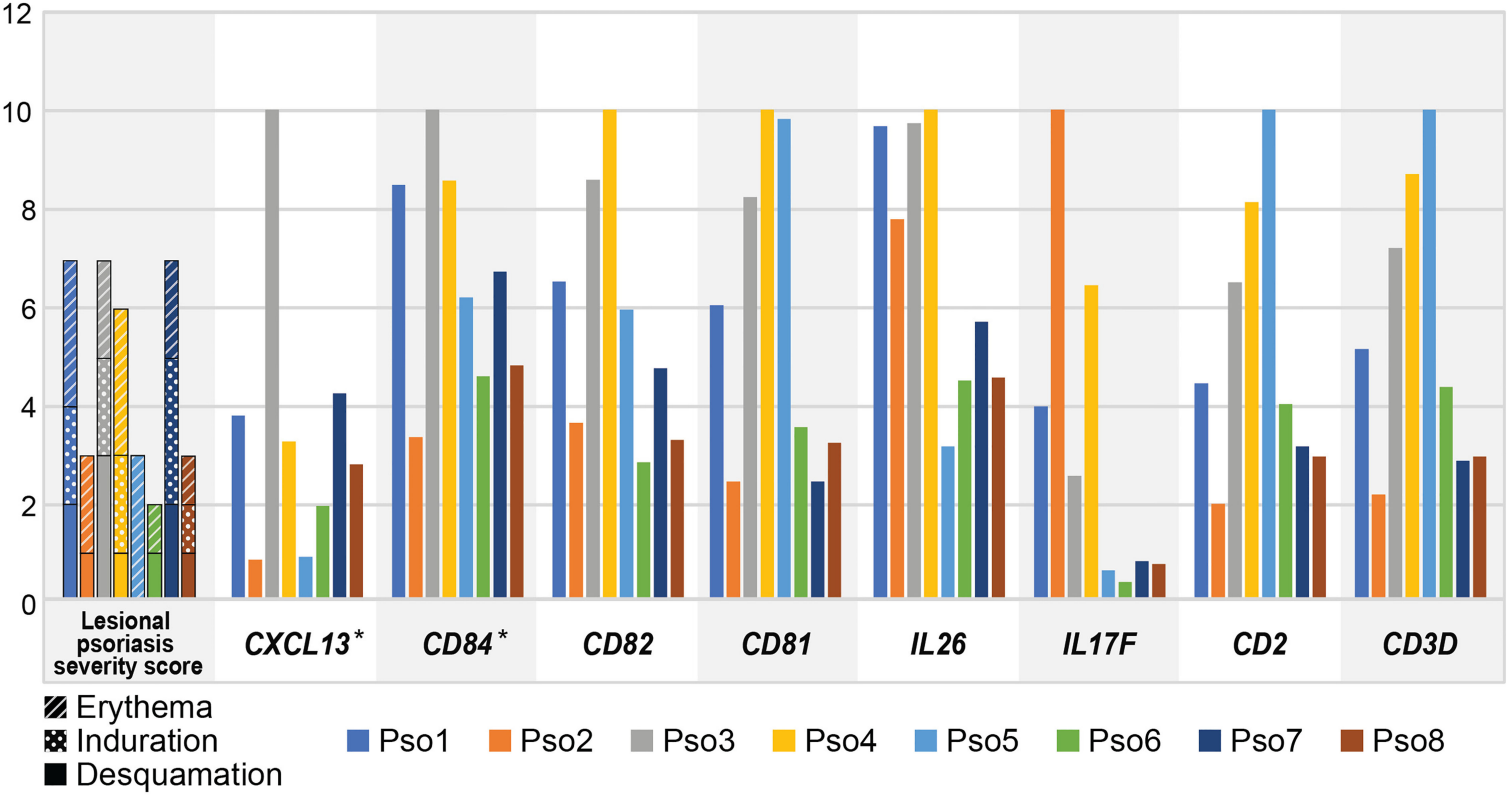
Inter-individual variation in lesional psoriasis severity score parallels that of *CXCL13* and *CD84*. Leftmost graph shows severity scores for biopsied lesion for each patient. Subsequent graphs display deduced number of cells with positive expression for each gene, as a percentage of the maximum number of positive cells in any sample, multiplied by 1 x 10^3^ (*i.e.* normalized to 10). Significant correlations for *CXCL13* and *CD84* are denoted by asterisks.

We then assessed Spearman correlation of T cell expression of all 16 immune cell DEGs with lesion-specific severity score. Three of these genes correlated strongly with lesion-specific scores: a single gene coefficient of 0.851 for *CXCL13*, and *IKZF4* (*p* = 7.3 x 10^-3^) and 0.801 for *CD84* (*p* = 1.7 x 10^-2^) (Bonferroni unadjusted, eight selected genes displayed in **Figure 3**; **Supplementary Table 5**). When the patient-level PASI score was used as an alternative comparator, none of the 16 immune genes showed significant correlations at unadjusted *p* values.

### Psoriatic CD45^+^ Cells Show Programmatic Activation of Mitotic Cell Division, Chromatin Remodeling, CDC42 Signaling, and Leukocyte Activation

**Figure 4:**
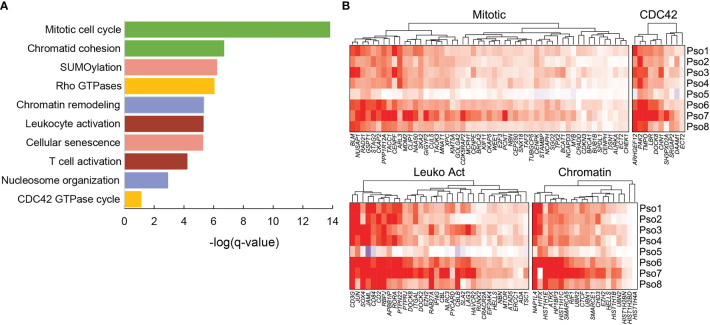
Significant functional associations for the 662 genes significantly elevated in psoriasis samples compared to grouped healthy controls in Trm2. **(A)** Ten example classifications are shown, with functions such as immune cell activation, mitotic cell division, and cytoskeletal reorganization. **(B)** Heatmaps visually represent average log2FC between individual psoriasis samples and normal controls using ComplexHeatmap (18). Heterogeneity is detected between patients, most prominently the dampened amplitude of transcript abnormalities in Patient 5, who was on systemic tacrolimus and mycophenolate at time of biopsy.

We next asked how functionally related groups of genes activated during psoriatic inflammation might vary in expression from patient to patient. We first applied the Metascape analysis package to detect overrepresentation of Gene Ontology and Reactome functional categories in the 662 genes significantly elevated (logFC > 0.4) in psoriatic skin-resident memory cells (Trm2), compared to healthy, controls, identifying 316 functional categories with a log (*q* value) < -2 (**Supplementary Table 6**; **Supplementary Data 2**). Statistically significant functional classes, included expected categories such as mitotic cell division and leukocyte activation (21 members, log (*q* value) < -9.97), but also highlighted the role of cytoskeletal reorganization (CDC42 signaling) and chromatin remodeling (**Figure 4A**). For example, *ARHGEF12* selectively regulates RhoA subfamily GTPases to coordinate cell migration and invasion (19), while *PAK2* influences actin cytoskeleton reorganization (20). *DOCK8* deficiencies impair immune cell migration in both the innate and adaptive immune system (21). Changes in psoriatic Trm also include elevated transcripts levels of the linker histone *H1FX* (22), histone chaperone *NAP1L4* (23), and the chromatin-modifying enzyme *SMARCA5* (24). **Figure 4B** globally displays psoriasis Trm2 abnormalities in these four programs on a per-patient level.

Considerable patient-specific fluctuations in these functionally related gene sets were easily appreciable. Most obviously, patient 5, the lone patient with psoriasis who was under systemic immunosuppression (mycophenolate and cyclosporine for a liver transplant) showed substantial attenuation of all these programs, corresponding to the lowest lesional psoriasis severity score (**Supplementary Table 1**). We sought to systematically assess these correlations between expression and phenotype, first averaging transcriptional log2FC for all genes in each of the four individual functional programs. Average scores for all four programs showed positive correlation with lesional severity score: CDC42 cytoskeletal reorganization at a Spearman rho value of 0.57, cell division at 0.55, chromatin reorganization at 0.48, and leukocyte activation at 0.36. None of these associations reached statistical significance, likely a factor of our limited sample size. However, in each pathway, activation parallels severity of individual lesions, revealing a potential source of some proportion of clinical heterogeneity.

### Psoriasis Single Cells Expressing High Levels of Pathogenic Cytokines Display Elevated T Cell Activation and Cytoskeletal Reorganization Genes

While pervasive elevation of transcripts regulating mitosis or CDC42-centric functional reorganization coincided with induction of pathogenic cytokines in the Trm compartment, we were uncertain whether these programs were related on the single-cell level. In one model, members of these programs might simply be stochastically elevated in any given, pathogenically IL23-polarized single T cell. Alternatively, we hypothesized that some of these transcriptional programs could be shared on the single-cell level, a pattern that could impact approaches to therapeutic targeting. For example, if the single T cells most likely to express Th17 cytokines also showed robust reprogramming of cytoskeleton genes, strategies restraining actin reorganization might impede the mobility and infiltration of the most pathogenic skin-resident T cells.

**Figure 5:**
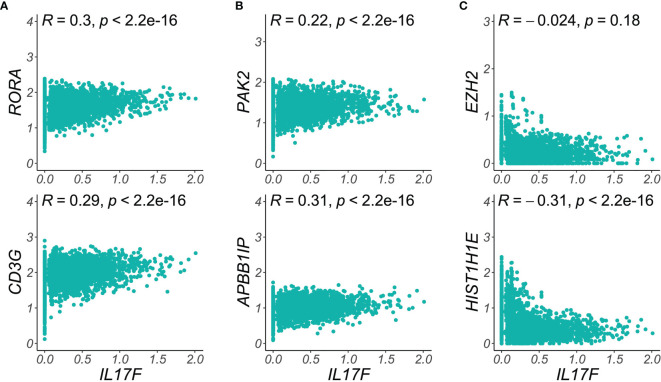
Single-cell correlations and anti-correlations between functional class transcripts and *IL17F* expression. **(A)** T cell activation markers like *RORA* and *CD3G* are elevated in the highest *IL17F* expressing cells, **(B)** Key cytoskeletal reorganization transcripts (*PAK2, APBB1IB*) are most elevated in the single skin-resident T cells expressing maximal psoriatic inflammatory mediators. **(C)** Chromatin remodeling transcripts (*EZH2*, *HIST1H1E*), are elevated in the lowest *IL17F* expression cells, suggesting a distinct, pathologic cell population in psoriatic Trm. Density plots show imputed single cell expression of T cell activation, chromatin remodeling, or cytoskeletal transcripts (*y*-axis) vs. *IL17F* (*x*-axis). Dots represent single Trm2 cells (psoriasis samples).

We therefore calculated the Pearson correlation coefficients for expression of pathogenic cytokines in single Trm2 cells against those of genes in our cytoskeletal and secretory classes, finding striking instances of both positive and negative correlation (**Figure 5**). For example, **Figure 5A** shows positive correlation of the *RORA* transcription factor with *IL17F* expression, as would be expected given its role in Th17 programming (R = 0.3, *p* = 2.2 x 10^-16^) (25), as well as for the TCR component *CD3G* (R = 0.29, *p* = 2.2 x 10^-16^). Similarly, in **Figure 5B**, the cytoskeletal re-organization genes *PAK2* and *APBB1IP* robustly positively correlate with *IL17F* expression in single skin-resident memory T cells, supporting a model in which more highly pathogenically activated cells are also more motile and capable of tissue infiltration. In sharp contrast, the single cells expressing maximum *IL17F* and those expressing elevated levels of a number of chromatin-modifying transcripts are negatively correlated, for example, a R of -0.30 for *HIST1H1E* (**Figure 5C**). Such instances of mutual exclusivity suggest the presence of a second, abnormal, non-Th17 population within psoriatic Trm, whose influence on disease state is yet undetermined. A comprehensive single-cell correlation table in Trm2 for *IL17F*, *CXCL13*, and *IL26* is available in **Supplementary Table 7**.

## Discussion

While a vast landscape of transcriptional abnormalities in immune and stromal cell types characterizes chronic inflammatory skin disease (26, 27), clinical improvement following inhibition of the IL12/23 pathway or blockade of IL17 isoforms validates the central role of psoriatic T cells. Our single-cell profiles of 8 psoriasis samples, along with normal controls, begin to illuminate patient-specific variation of transcriptional abnormalities in psoriatic Trm (**Figure 2**). Psoriasis DEGs with greater than average patient-level variance, reflected in higher dispersion scores, include the most recognizable Th17 cytokines such as *IL17F* and *IL26*, recently implicated inflammatory psoriatic mediators such as *CXCL13* (13), and genes orchestrating cell division in mitotically active T cells. Such psoriasis DEGs with higher dispersion scores may represent sources of patient-specific phenotypic and clinical variability, such as lesion intensity or anatomic distribution.

Expression of *CXCL13* and *CD84* correlated significantly with lesional severity score in our study, a predicate for further investigation as sources or important associations of disease state. Our data adds to increasing evidence that *CXCL13* represents a particularly Th17-specific abnormality (8) and positively associates with psoriasis severity (13, 28), nominating it as a clinically useful biomarker for cutaneous disease. *CD84* is a known T cell activation marker, genetic variants of which have been associated with response in psoriasis to TNF blockade (29). Interestingly, *IL17F* expression in our series correlated poorly with lesional severity score but was highly elevated in scalp psoriasis, suggesting it might show anatomic specificity in more highly powered studies. This finding comports with an earlier scRNA-seq report that Th17 cytokine expression and overall inflammatory state is surprisingly prominent in healthy scalp cells (27). The key constraint of our study is patient number, limited by the current costs of scRNA-seq. It is very likely additional such correlations will reach significance as these approaches are extended to larger data sets.

Conversely, psoriasis-specific DEGs harboring lower dispersion scores may be more suitable for broader screening to identify psoriasis-like molecular profiles, a feature that may help direct biological treatment for the subset of rashes demonstrating both psoriasiform and spongiotic histopathology (30). Within the set of psoriasis-specific skin-resident DEGs that are overexpressed relative to analogous T cells in atopic dermatitis, examples of such lower variance Th17 biomarkers include the GTPase-activator *CHN1* (0.069) and *PTMS* (0.077).

We also undertook a systematic search of coordinated functional derangements in skin-resident T cells, based on the increased resolution afforded by single-cell transcriptomics. Such groups of pathologic transcriptional alternations may function as quantitative traits, collectively modifying disease phenotype beyond the impact of dysregulated single genes. Applying this method, we detected not only expected elevations in inflammatory signalling and cell division, but also global increases in pathways coordinating CDC42-centric cytoskeletal reorganization and chromatin remodeling. In one sense, broad alterations in these programs are not surprising, given the profound changes in cell polarity and motility that accompany T cell activation. However, this is the first report describing recurrent upregulation of dozens of these transcripts in pathologically inflamed T cells. All 8 patients in our series show abnormalities in these programs (**Figure 4**), whose elevation trends with lesional psoriasis severity scores, supporting a role in the pathogenicity of skin inflammation.

We also show that single T cells expressing the highest levels of psoriatic inflammatory mediators such as *IL17F* are markedly enriched for cytoskeletal remodeling transcripts, suggesting such programs may facilitate tissue infiltration and cytokine secretion. Combination therapeutic approaches targeting both Th17 polarization and cytoskeletal activity may thus synergistically target a common population of particularly pathogenic skin T cells. We also find that certain chromatin remodeling DEGs peak in single T cells distinct from those maximally expressing *IL17F*, indicating these data can also identify additional, abnormally reprogrammed subpopulations within the Trm compartment.

In summary, the analyses presented here describe a suite of quantitative approaches to evaluate high-resolution transcriptional variation between psoriasis patients. The most distinguishing abnormalities are identified in skin-resident T cells, and even our limited test set identifies credible associations between specific genes and lesion phenotype. Greater numbers of scRNA-seq datasets are now becoming publicly accessible. Systematic identification of such instances of inter-individual molecular heterogeneity will make it possible to test clinically predictive associations for both single genes and aggregate molecular disease signatures.

## Data Availability Statement

Sequencing BAM files are deposited at the European Genome-Phenome Archive (EGA) under accession number EGAS00001005271. The processed Seurat object with cell identities is available at Zenodo (https://zenodo.org/record/6529821#.Ynh_aoxBwuW).

## Ethics Statement

The studies involving human participants were reviewed and approved by University of California, San Francisco Institutional Review Board. The patients/participants provided their written informed consent to participate in this study.

## Author Contributions

YL, JC, EP, and RC designed the study. EK, JC, and RC supervised sample collection and processing. YL, CC, AH, and AS performed sample preparation and analysis. HW, MT, CC, YS, and EP performed computational analysis. JN performed histopathology analysis. YL, HW, EP, JC, and RC wrote the original manuscript with contributions from CC, JN, and AH. All authors contributed to the article and approved the submitted version.

## Funding

This study received funding from Sun Pharmaceutical Industries. The funder was not involved in the study design, collection, analysis, and interpretation of data, the writing of this article or the decision to submit it for publication. JC and RC are funded in part by grants from the National Institute of Arthritis and Musculoskeletal and Skin Diseases of the National Institutes of Health K08AR067243, the National Psoriasis Foundation, and a LEO foundation grant.

## Conflict of Interest

JC and RC receive support (research grants to their institution) from LEO Pharmaceuticals, Sanofi, and Pfizer.

The remaining authors declare that the research was conducted in the absence of any commercial or financial relationships that could be construed as a potential conflict of interest.

## Publisher’s Note

All claims expressed in this article are solely those of the authors and do not necessarily represent those of their affiliated organizations, or those of the publisher, the editors and the reviewers. Any product that may be evaluated in this article, or claim that may be made by its manufacturer, is not guaranteed or endorsed by the publisher.

## Supplementary Material

Supplementary Table 1 Clinical Characteristics of Psoriasis Patients.

Supplementary Table 2 Cell Type Marker Transcripts (Seurat-derived).

Supplementary Table 3 Trm2 Pseudo-bulk DEGs Normal Controls vs. Psoriasis.

Supplementary Table 4 Deduced Cell Number Expression for each Gene.

Supplementary Table 5 Correlation of Gene Expressing Cells with Lesional Psoriasis Severity Scores.

Supplementary Table 6 Metascape Functional Analysis of Trm2 Psoriasis DEGs.

Supplementary Table 7 Single-cell gene correlations (non-imputed) in Trm2 for *IL17F*, *CXCL13*, and *IL26*.

Supplementary Data Sheet 1 uMAP representation of donors, showing high representation for each sample in key immune cell classes.

Supplementary Data Sheet 2 Box plots of selected genes from Trm2 DEG enriched functional classes on a per-sample basis.
