# Supplementary figures and images for: Defining Patient-Level Molecular Heterogeneity in Psoriasis Vulgaris Based on Single-Cell Transcriptomics
Source: Front Immunol. 2022 Jul 26;13:842651. doi: 10.3389/fimmu.2022.842651 (PMC9360479; doi:10.3389/fimmu.2022.842651)

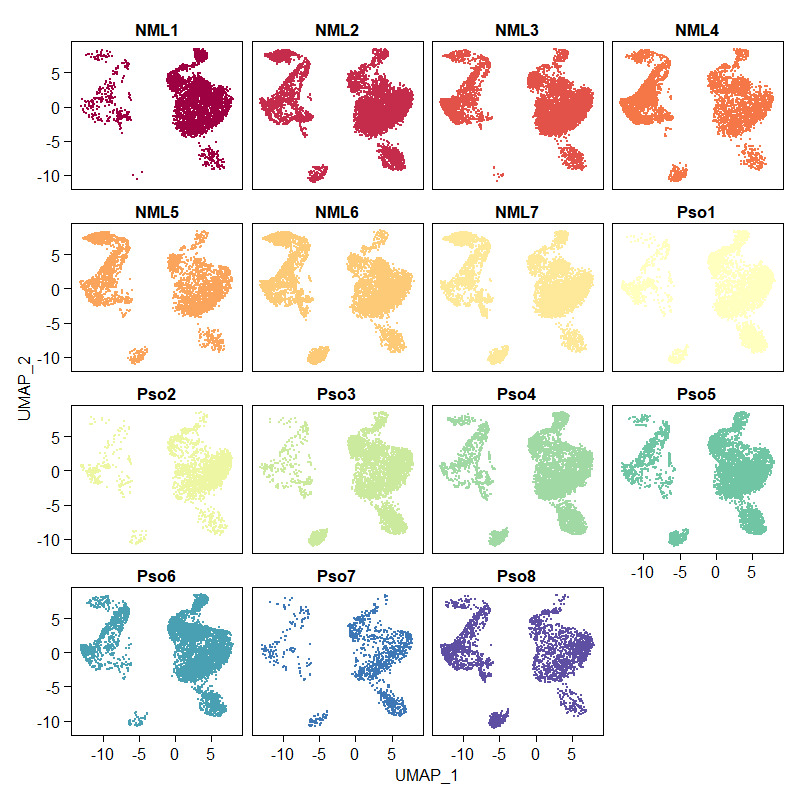

Supplement: Supplementary Data Sheet 1 — uMAP representation of donors, showing high representation for each sample in key immune cell classes. [file Image_1.jpeg]

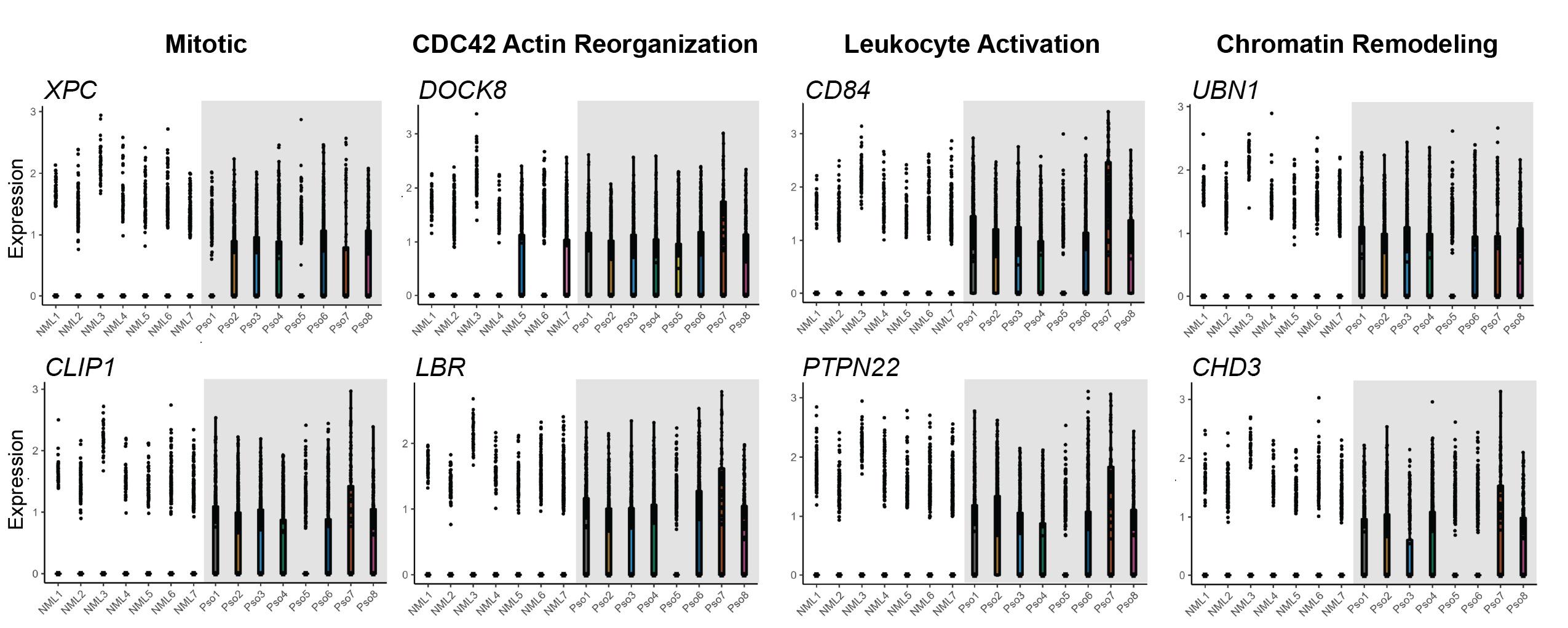

Supplement: Supplementary Data Sheet 2 — Box plots of selected genes from Trm2 DEG enriched functional classes on a per-sample basis. [file Image_2.jpeg]
